# Epithelioid Hemangioendothelioma With a Novel Chromosomal Deletion and an Aggressive Clinical Course: A Case Report and Literature Review

**DOI:** 10.1155/crom/3387413

**Published:** 2026-02-27

**Authors:** Danielle C. Thor, Nowair Hussain, Ada Baisre de Leon

**Affiliations:** ^1^ Jefferson Health-New, Stratford, New Jersey, USA; ^2^ Overlook Medical Center, Summit, New Jersey, USA, atlantichealth.org; ^3^ Rutgers New Jersey Medical School, Newark, New Jersey, USA, rutgers.edu

**Keywords:** cervical radiculopathy, EHE, epithelioid hemangioendothelioma, hemangioendothelioma, hematology, oncology, pathology

## Abstract

Epithelioid hemangioendothelioma (EHE) is a rare neoplastic process arising from the endothelial lining of virtually any blood vessel, with varying degrees of metastatic spread. In the following case report, the clinical course and treatment stratification are detailed for a 59‐year‐old female who originally presented with cervical radiculopathy and was found to have aggressive‐type EHE refractory to multiple lines of therapy. In doing so, a novel deletion of the long arm of Chromosome 16 at Position 24, in all cells analyzed, ISCN Karyotype: 46,XX,del(16)(q24)[10] is also identified and may contribute to a more aggressive disease presentation. Ultimately, further scientific contribution is provided by this report to the otherwise limited understanding of this unique malignancy.

## 1. Introduction

Epithelioid hemangioendothelioma (EHE) is a rare neoplastic process arising from the endothelial lining of myriad blood vessels. EHE is categorized as a vascular sarcoma with an estimated annual incidence of one in one to three million individuals per year [1, 2]. The prognosis of EHE ranges from benign or asymptomatic disease to aggressive, symptomatic disease with diffuse metastatic potential [3]. These metastases typically spread to the liver, lungs, or bones, but can theoretically grow from all vascular structures [2, 4]. Symptomatic EHE most often presents with a painful, palpable mass in virtually any location with weight loss, and such upfront symptomatology typically confers a poorer prognosis [5, 6]. However, given the fact that most cases of aggressive EHE present metastatically due to low indexes of clinical suspicion at the time of diagnosis, its true presenting symptoms may vary.

Alongside this diverse presentation, EHE also maintains an unpredictable clinical course and prognosis. Its pathological behavior ranges anywhere from localized, indolent, and self‐limited incidence with potential for curative treatment to a high‐grade, aggressive sarcoma, with a survival expectancy between 1 and 11 months [5, 6]. Through this report, we intend to chronicle the management of an aggressive presentation of EHE from its presentation as a progressive cervical radiculopathy to its eventual management as a destructively metastatic disease refractory to several lines of therapy. We highlight a novel chromosomal deletion which may have conferred the aggressive course taken by this disease.

## 2. Case Description

A 59‐year‐old female with a past medical history of Bell’s Palsy presented to an outside institution with complaints of pain first thought to be musculoskeletal in origin. She described pain in her fourth upper left digit that spread to her remaining fingers and neck within the following month. She was evaluated at a pain management center and underwent EMG which suggested both C6 radiculopathy and carpal tunnel syndrome. Conservative management with naproxen, acetaminophen, diclofenac, and tizanidine provided minimal benefit. However, physical therapy, including hand/fine motor strengthening, range of motion exercises, and cervical traction exercises, provided significant relief, further supporting the original diagnosis.

Despite these perceived improvements, the patient′s pain worsened over the following months and expanded to involve the entire left upper extremity with new‐onset wrist drop. Magnetic resonance imaging (MRI) of the left brachial plexus was obtained and demonstrated a 3.4‐cm mass along the course of the left brachial plexus at the level of the proximal humeral diaphysis, as well as increased T2 signaling within the visualized proximal triceps muscle. An additional MRI of the left humerus was obtained which demonstrated an elongated, enhancing 6.4 cm mass encasing the patient′s entire neurovascular bundle at the proximal left humerus.

The patient was then referred to neurosurgery where evaluation for both metastatic disease via computerized tomography (CT) and nonneoplastic etiologies, such as sarcoidosis or infection, was nonrevealing. Given the continued high clinical suspicion of malignancy, the decision was made to biopsy the humeral lesion and send specimens for pathological review. Positron emission tomography with computerized tomography (PET‐CT) was obtained directly following the patient′s biopsy and demonstrated innumerable additional lesions within the patient′s left breast, lung, and inguinal regions. The patient was thereby referred to hematology/oncology for further management.

**Figure 1:**
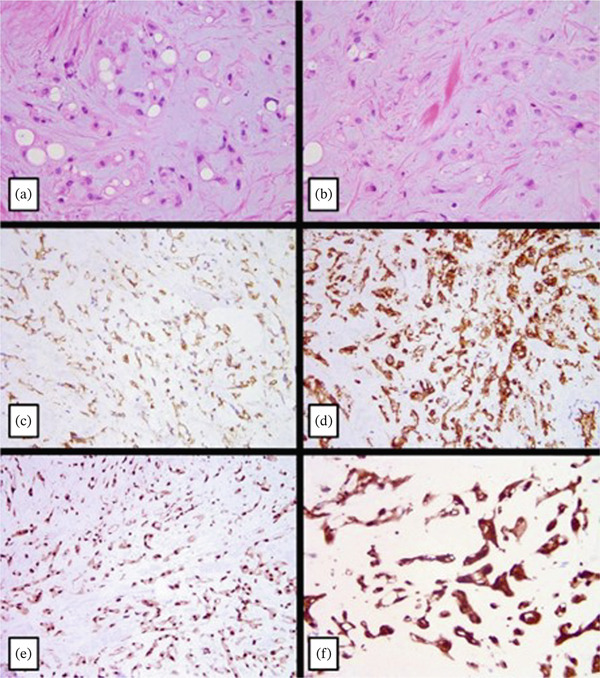
(a–f) Histopathological examination of the biopsy of the humerus lesion.

By the time of the patient′s oncological examination, the finalized pathology report was provided with a definitive diagnosis of EHE. Histopathological examination revealed the classical histology of EHE, with a mildly cellular neoplasm composed of spindle and epithelioid cells with eosinophilic cytoplasm and round‐to‐oval nuclei arranged in chords, nests, and single cells, in a myxoid to hyaline stromal matrix (Figure 1a,b). Several of the cells showed intracytoplasmic vacuoles which rarely contained red blood cells. The neoplastic cells diffusely expressed cytoplasmic CD31 (Figure 1c) and CD34 (Figure 1d) and nuclear expression of FLI‐1 (Figure 1e) and CAMTA‐1 (Figure 1f).

**Figure 2:**
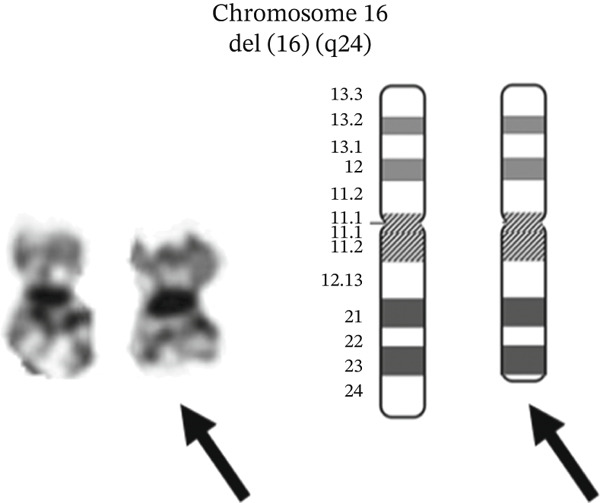
Chromosomal deletion map outlining the novel deletion on Chromosome 16, Karyotype: 46,XX,del (16)(q24) [10].

Chromosome analysis of 10 available metaphase cells showed a deletion of the long arm of Chromosome 16 at Position 24, in all cells, ISCN Karyotype: 46,XX,del(16)(q24)[10] (Figure 2), which is not a feature that has been previously described in EHE. The significance of this novel chromosomal deletion remained unclear throughout her disease course. Lastly, TFE3 gene on chromosome Xp11.22 was not rearranged in 60 interphase cells analyzed, by fluorescence in situ hybridization (FISH) using the TFE3 BA (Xp11.22) probe, ISCN Karyotype: nuc ish (TFE3x2, 60], very likely excluding the presence of a *YAP1::TFE3* fusion. Having sufficient evidence to confirm the diagnosis of EHE with *WWTR1::CAMTA1* fusion, other studies, for example, RNA fusion or copy number array studies, were not performed.

Given the extent of her disease, the patient was deemed a poor candidate for further surgical intervention and was instead started on daily pazopanib as per the most up‐to‐date National Comprehensive Cancer Network (NCCN) guidelines available at the time. However, the patient did not derive any significant clinical benefit and was later started on doxorubicin, of which she completed five cycles of treatment. A follow‐up CT of the chest, abdomen, and pelvis demonstrated mediastinal and hilar lymphadenopathy with new diffuse metastases to the liver, spleen, and bones. At this point, the patient was considered unlikely to respond to further chemotherapy but remained under consideration for palliative chemotherapy.

**Figure 3:**
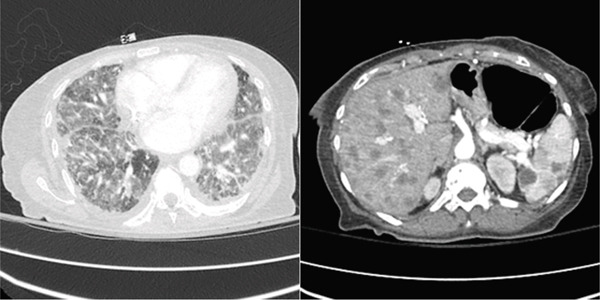
Key images from the repeat CT of the chest, abdomen, and pelvis demonstrating worsening lymphangitic spread of the patient′s EHE.

As a final effort to minimize her disease burden, the patient began treatment with docetaxel/gemcitabine but was unable to complete her first cycle due to two hospital admissions for acute hypoxic respiratory failure. Her initial admission resulted in stabilization and discharge with home oxygen; however, she was readmitted within several days of discharge for the same complaint. On this second admission, repeat CT imaging of the chest, abdomen, and pelvis was obtained and noted worsening of the lymphangitic spread of her disease (Figure 3). Her palliative chemotherapy regimen was inevitably discontinued, and after exhaustion of all inpatient stabilization efforts, a shared decision was reached between the patient, her family, and her care team to pursue home hospice. The patient passed away at home with her family within a month following discharge.

## 3. Discussion

In this case report, we present an aggressive, symptomatic EHE characterized by a novel chromosomal abnormality and a poor response to systemic therapy. As a rare malignancy with 50%–75% of patients presenting asymptomatically, EHE is often misdiagnosed or underdiagnosed. Its clinical presentation is highly variable, although blood vessels represent the primary site of origin in nearly half of confirmed cases [2]. Because of this, diligent histopathologic evaluation of suspicious lesions remains essential for accurate diagnosis, prognostication, and treatment planning [1]. Early stratification of asymptomatic versus symptomatic disease is particularly important, as asymptomatic EHE is associated with a median survival exceeding 10 years, whereas symptomatic disease confers a median survival of less than 1 year [5].

Two distinct molecular subtypes of EHE are recognized by the WHO Classification of Tumors [7]. EHE with WWTR1::CAMTA1 fusion accounts for approximately 90% of cases, while EHE with YAP1::TFE3 fusion represents the remaining ~10% [6, 8]. Tumors harboring the WWTR1::CAMTA1 fusion exhibit characteristic morphologic features, including epithelioid cells with eosinophilic cytoplasm arranged in cords, nests, and single cells within a myxoid to hyaline stromal matrix, along with strong nuclear CAMTA‐1 expression, as seen in this case (Figure 1). In contrast, YAP1::TFE3–rearranged tumors demonstrate abundant eosinophilic cytoplasm, nested growth patterns, and diffuse TFE‐3 expression, although these findings are not entirely specific and often necessitate confirmatory testing such as FISH. Greater nuclear atypia, necrosis, and mitotic activity exceeding two mitoses per 10 high‐power fields have all been associated with more aggressive clinical behavior [9].

In the case presented here, a novel chromosomal deletion, del(16)(q24)[10], was identified in the setting of otherwise typical histopathologic features of aggressive EHE. Deletions involving Chromosome 16q have been associated with poor prognosis in several malignancies, including endometrial cancer, prostate cancer, and multiple myeloma [10–12], while paradoxically correlating with improved outcomes in select lower grade breast cancers [13], underscoring the context‐dependent biological effects of this alteration. Although multiple gene aberrations are described in the most recent NCCN guidelines for EHE, deletions of Chromosome 16q, including del(16)(q24)[10], have not been previously reported [14]. Given the uniformly aggressive clinical course and lack of response to therapy in this case, it is plausible that the del(16)(q24)[10] deletion may either contribute to or serve as a marker of poorer prognosis in EHE.

Current treatment options for localized EHE emphasize surgical resection with curative intent when feasible, with reported cure rates of 70%–80% when negative margins are achieved [15, 16]. Patients with unresectable disease or positive margins may derive benefit from radiation therapy or, less commonly, percutaneous ablation, *trans*‐arterial chemoembolization, radioembolization, or isolated limb perfusion [9, 14]. Conversely, extensive surgical approaches such as liver transplantation have demonstrated limited benefit in hepatically confined EHE [11], though image‐guided microwave ablation has emerged as a promising alternative in select patients [17].

For patients with advanced or metastatic EHE, systemic therapy is largely palliative and guided by disease burden, symptom progression, and organ dysfunction [9, 14]. To date, no systemic agent has demonstrated consistent efficacy. mTOR inhibitors have shown the greatest clinical benefit, with progression‐free and overall survival approaching 1–2 years [9]. Other treatment options include antiangiogenic agents such as VEGFR tyrosine kinase inhibitors and cytotoxic chemotherapy [18, 19], while immunomodulatory agents such as lenalidomide and sirolimus have demonstrated modest benefit in small series [20, 21]. Emerging therapies, including MEK inhibition with trametinib, show early promise but require further validation [22]. In the absence of definitive treatment paradigms, management of advanced EHE necessitates an individualized approach, ideally involving multidisciplinary review at a specialized sarcoma center.

Although limited to a single patient, this case is notable for documenting an aggressive presentation of EHE associated with a previously unreported Chromosome 16q deletion. It raises the possibility that secondary cytogenetic abnormalities, beyond canonical fusion events, may identify a subset of EHE patients at increased risk for aggressive disease and treatment resistance. As such, this report contributes to the limited existing literature on EHE and supports further investigation into the prognostic significance of copy number alterations in this rare malignancy.

## 4. Conclusion

This case report details a patient with an aggressive presentation of an otherwise rare type of sarcoma with a unique chromosomal deletion found during pathological review. In doing so, further contributions are made to the understanding of the EHE disease process, clinical course, and treatment stratification. Continued opportunity for the study of EHE is encouraged, including further correlation between the presence of this Chromosome 16q deletion and aggressive disease.

## Author Contributions

D.C.T. and N.H. participated in the care of the patient and wrote the manuscript text. A.B.d. provided pathological analysis and prepared Figures 1 and 2. All authors reviewed the manuscript.

## Funding

No funding was received for this manuscript.

## Disclosure

An overview of this case was presented at the Oncology Society of New Jersey’s 2024 Annual Spring Meeting [23] and was awarded second place in the resident poster presentation competition.

## Ethics Statement

Informed consent for participation was obtained from the patient and documented in a HIPAA‐compliant manner prior to the preparation of this manuscript.

## Consent

Informed consent for publication was obtained from the patient and documented in a HIPAA‐compliant manner prior to the preparation of this manuscript.

## Conflicts of Interest

The authors declare no conflicts of interest.

## Data Availability

The data that support the findings of this study are available on request from the corresponding author. The data are not publicly available due to privacy or ethical restrictions.
